# Supplementary material for: Sex-Specific Catabolic Metabolism Alterations in the Critically Ill following High Dose Vitamin D
Source: Metabolites. 2022 Feb 25;12(3):207. doi: 10.3390/metabo12030207 (PMC8953844; doi:10.3390/metabo12030207)
Supplement: Supplementary file 1 [file metabolites-12-00207-s001.zip › Methods S1.pdf]

Sex-specific catabolic metabolism alterations in the critically ill following High Dose Vitamin D

Sowmya Chary, MBBS, Karin Amrein, MD, MSc, Jessica A. Lasky-Su, ScD, Sherif H. Mahmoud BSc (Pharm), MSc, PhD, Kenneth B. Christopher, MD, SM

Supplemental Methods

Trial Details: The VITdAL-ICU trial randomized 475 critically ill adult subjects with 25(OH)D ≤ 20 ng/mL to vitamin D_3 or placebo given orally or via nasogastric tube once at a dose of 540,000 IU followed by 90,000 IU monthly [1]. Patients admitted to University Hospital Graz in Southeast Austria in one of 5 Medical and Surgical Intensive Care Units (ICU), 18 years or older, with an expected ICU stay of ≥ 48 hours, with a 25(OH)D level of 20 ng/mL or lower were eligible for study participation. Patients were excluded from the trial who met any of the following criteria: severely impaired gastrointestinal function; other trial participation, including previous participation in the pilot trial; pregnant or lactating women; hypercalcemia (total calcium of >10.6 mg/dL or ionized serum calcium of >5.4 mg/dL); tuberculosis; sarcoidosis; nephrolithiasis within the prior year; and patients not deemed suitable for study participation (i.e., psychiatric disease, living remotely from the clinic, or prisoner status).

VITdAL-ICU trial subjects were randomized 1:1 with randomization block size of 8 stratified via ICU type and sex. The primary trial study outcome was length of hospital stay. Secondary outcomes included 28-day mortality, hospital mortality, 6-month

mortality, length of ICU stay and 25(OH)D levels at day 0, 3 and 7. Blood samples were collected on days 0 (pre-randomization), 3 and 7. Plasma was fractionated, aliquoted and stored at -70°C. 453 trial subjects had frozen plasma available for analysis. At VITdAL-ICU trial enrollment, written informed consent was obtained, if possible, directly from the patient or from a legal surrogate [1]. Consent included permission for plasma specimens to be saved for future research studies. The post-hoc study research protocol was approved by the Mass General Brigham Human Research Committee Institutional Review Board at the Brigham and Women's Hospital.

Clinical trial data utilized included age, sex, admission diagnosis category, Charlson Comorbidity Index [2], baseline 25(OH)D, intervention status (placebo vs high dose vitamin D3), absolute change in 25(OH)D level at day 3 relative to day 0 and the Simplified Acute Physiology Score (SAPS) II [3] at day 0. Admission diagnosis category is determined at ICU admission by trial investigators and includes Neurosurgery, Cardiac surgery, Cardiovascular, Gastrointestinal/liver, Hematologic/Oncology/ Metabolic, Neurologic, Other non-operative, Other operative, Renal, Respiratory, Sepsis/infectious, Thoracic surgery, Transplantation, Trauma and Vascular.

Sample Preparation: VITdAL-ICU trial subject plasma aliquots were shipped on dry-ice to Metabolon, Inc. Following receipt, the frozen plasma samples were immediately stored at -80°C. To generate metabolomic data, a total of 1215 plasma samples from 428 subjects at day 0, 413 subjects at day 3 and 374 subjects at day 7 were prepared

and analyzed. Plasma sample preparation was performed with the automated MicroLab STAR® Liquid Handling system (Hamilton Company, NV, USA). Before extraction, samples were fortified with recovery standards for quality control (QC) purposes. To remove protein, dissociate small molecules bound to protein or trapped in the precipitated protein matrix, and to recover chemically diverse metabolites, proteins were precipitated with methanol via 2 minutes of robust shaking (GenoGrinder 2000 SPEX SamplePrep, NJ, USA) and subsequent centrifugation. The resulting extract was divided into five fractions: two for analysis by two separate reverse phase (RP)/UPLC-MS/MS methods with positive ion mode electrospray ionization (ESI), one for analysis by RP/UPLC-MS/MS with negative ion mode ESI, one for analysis by HILIC/UPLC-MS/MS with negative ion mode ESI, and one sample was reserved for backup. Samples were placed on a TurboVap® (Zymark, MA, USA) to remove the organic solvent and stored overnight under nitrogen before preparation for analysis.

Quality Assurance (QA) and Quality Control (QC): Several types of controls were utilized with the plasma samples analysis: a pooled matrix sample generated by taking a small volume of each experimental sample served as a technical replicate throughout the data set [4]; extracted water samples served as process blanks [5]; and a cocktail of QC standards that were carefully chosen not to interfere with the measurement of endogenous compounds were spiked into every analyzed sample [6], allowed instrument performance monitoring and aided chromatographic alignment. Instrument variability was determined by calculating the median relative standard deviation (RSD) for the standards that were added to each sample prior to injection into the mass

spectrometers [7]. Overall process variability was determined by calculating the median RSD for all endogenous metabolites (i.e., non-instrument standards) present in 100% of the pooled matrix samples. Experimental samples were randomized across the platform run with QC samples spaced evenly among the injections.

Ultrahigh Performance Liquid Chromatography-Tandem Mass Spectroscopy (UPLC-MS/MS): All methods utilized a Waters ACQUITY ultra-performance liquid chromatography (UPLC) (Waters, MA, USA) and for untargeted lipidomic analysis a Thermo Scientific Q Exactive™ high resolution/accurate mass spectrometer interfaced with a heated electrospray ionization (HESI-II) source and Orbitrap™ mass analyzer operated at 35,000 mass resolution (ThermoFisher Scientific, MA, USA) [8]. The sample extract was dried then reconstituted in solvents compatible to each of the four methods. Each reconstitution solvent contained a series of standards at fixed concentrations to ensure injection and chromatographic consistency. One aliquot was analyzed using acidic positive ion conditions, chromatographically optimized for more hydrophilic compounds. In this method, the extract was gradient eluted from a C18 column (Waters UPLC BEH C18-2.1x100 mm, 1.7 µm) using water and methanol, containing 0.05% perfluoropentanoic acid (PFPA) and 0.1% formic acid (FA). Another aliquot was also analyzed using acidic positive ion conditions, however it was chromatographically optimized for more hydrophobic compounds [9,10]. In this method, the extract was gradient eluted from the same afore mentioned C18 column using methanol, acetonitrile, water, 0.05% PFPA and 0.01% FA and was operated at an overall higher organic content. Another aliquot was analyzed using basic negative ion optimized

conditions using a separate dedicated C18 column. The basic extracts were gradient eluted from the column using methanol and water, however with 6.5mM Ammonium Bicarbonate at pH 8. The fourth aliquot was analyzed via negative ionization following elution from a HILIC column (Waters UPLC BEH Amide 2.1x150 mm, 1.7 µm) using a gradient consisting of water and acetonitrile with 10mM Ammonium Formate, pH 10.8. The MS analysis alternated between MS and data-dependent MS^n scans using dynamic exclusion [11]. The scan range for both ionization modes was 70–1000 *m/z* [12].

Data Extraction and Compound Identification: Raw data was extracted, peak-identified and QC processed using Metabolon's hardware and software. Compounds were identified by comparison to library entries of purified standards or recurrent unknown entities. Metabolon maintains a library based on authenticated standards that contains the retention time/index (RI), mass-to-charge ratio (*m/z*), and chromatographic data (including MS/MS spectral data) on all molecules present in the library. Furthermore, biochemical identifications are based on three criteria: retention index within a narrow RI window of the proposed identification, accurate mass match to the library +/- 10 ppm, and the MS/MS forward and reverse scores between the experimental data and authentic standards [13]. The MS/MS scores are based on a comparison of the ions present in the experimental spectrum to the ions present in the library spectrum. While there may be similarities between these molecules based on one of these factors, the use of all three data points can be utilized to distinguish and differentiate biochemicals [14]. More than 3300 commercially available purified standard compounds have been acquired and registered into the Metabolon Laboratory

Information Management System (LIMS) system for analysis on all platforms for determination of their analytical characteristics. The identification level reported in our tables follows the criteria described by Sumner et al. [15]. Level 1 is a validated identification which confirms a structure with a minimum of two independent and orthogonal data from a pure reference standard under identical analytical conditions. Predictive or externally acquired structure evidence when a reference standard does not exist, (i.e. MS/MS data, exhibiting diagnostic fragments or neutral losses consistent with a specific structure) is a putative identification (Level 2) [16]. Compounds labelled with "*" have identification Level 2. If no label is applied, the identification Level is 1. Compounds labelled with "( )" or "[ ]" indicate a structural isomer of another compound in the spectral library; for example, a steroid that may be sulfated at one of several positions that are indistinguishable by the mass spectrometry data or a diacylglycerol for which more than one stereospecific molecule exists. For the Acylcarnitine sub pathway: a capital C is followed by the number of carbons within the fatty acyl group attached to the carnitine. A colon followed by a number is one or more unsaturated carbons in the acylcarnitine ester (i.e. C10:1 is a monounsaturated C10 acylcarnitine). DC following the carbon number is a dicarboxylic acylcarnitine. Acylcarnitines are classified by the number of carbon atoms in the acyl group chain: short-chain acylcarnitines C2 to C7; medium-chain acylcarnitines C8 to C14; long-chain acylcarnitines C16 – C26 [17]. A summary of all 983 metabolites identified is present in Data S1.

Curation: A variety of curation procedures were carried out to ensure that a high quality data set was made available for statistical analysis and data interpretation. The

QC and curation processes were designed to ensure accurate and consistent identification of true chemical entities, and to remove those representing system artifacts, mis-assignments, and background noise. Metabolon data analysts use proprietary visualization and interpretation software to confirm the consistency of peak identification among the various samples. Library matches for each compound were checked for each sample and corrected if necessary.

Metabolite Quantification and Data Normalization: Peaks were quantified using total spectral area (area under the curve) [18-20]. Metabolite quantitation or abundance is defined as the total ion count for the given mass-to-charge ratio (*m/z*) assigned to the particular metabolite [21]. Specifically, metabolite quantitation is determined using extracted ion chromatograms by focusing the narrow mass window on the theoretical *m/z* value of the individual metabolite of interest and eliminating overlapping isobaric signals with maintenance of the mass accuracy during the acquisition [22-26]. A data normalization step was performed to correct variation resulting from instrument inter-day tuning differences. Each compound was corrected in run-day blocks by registering the medians to equal one (1.00) and normalizing each data point proportionately.

Pharmacokinetics: For determination of the pharmacokinetics of oral vitamin D_3 we utilized serum 25(OH)D levels, a marker of systemic vitamin D status [27]. Serum 25(OH)D were measured by chemiluminescence assay (IDS-iSYS, Immunodiagnostic Systems) with assay coefficients of variation for control material of 13.4% at 13 ng/mL,

10% at 31 ng/mL, and 9.4% at 64 ng/mL [1]. For pharmacokinetics evaluation, the area under the plasma concentration–time curve from vitamin D_3 dosing to day 7 (AUC0-7d) was calculated using the linear trapezoidal method. Patients with missing 25(OH)D levels on day 3 or 7 and those that received placebo were excluded from the pharmacokinetics evaluation. AUC normalized to vitamin D_3 dose and body weight (AUCnorm) was calculated by dividing AUC 0-7d by dose in IU per kg body weight. Median AUCnorm and serum 25(OH)D levels on days 0, 3 and 7 values were compared between males and females.

Statistical Analyses: Determination of the changes in relative concentrations of metabolites was first suggested as a strategy to define the metabolome in 1998 [28]. Metabolomic profiling identified 983 metabolites [29,30]. Metabolomic data underwent a cube root transformation followed by Pareto scaling to generate data that were on the same scale and followed an approximate normal distribution [31,32].

Based on our previous metabolomics analysis of the VITdAL-ICU trial [29] , we considered a response to high dose oral vitamin D_3 as an absolute increase in 25(OH)D ≥ 7.5 ng/ml from day 0 to day 3. For a sex-stratified analysis of day 0 data, Student's t test was used to determine the significance of each metabolite between vitamin D_3 response groups [25(OH)D < or ≥ 7.5 ng/ml from day 0 to day 3] using MetaboAnalyst in women and in men [33]. To identify all significant associations we utilized multiple testing correction based on the Benjamini-Hochberg procedure to adjust the false discovery rate (FDR) to 0.10 producing a q-value [34]. Day 0 data was also analyzed using

orthogonal partial least square-discriminant analysis (OPLS-DA), also known as orthogonal projections to latent structures discriminant analysis, a supervised method to assess the significance of classification discrimination (SIMCA 15.0 Umetrics, Umea, Sweden).

We utilized the OPLS-DA approach to find what were the metabolomic differences at baseline (day 0) between patients who did and did not respond to high dose vitamin D_3. We analyzed male and female subjects separately to determine if such differences in metabolites at baseline were sex-specific. OPLS-DA was performed to relate the X data to the Y response [35,36]. In our study, the X are the metabolites at day 0 and the Y is the intervention (vitamin D_3 response groups [25(OH)D < or ≥ 7.5 ng/ml from day 0 to day 3]). We assessed the OPLS-DA model quality via the variation of X explained by the model (R2X(cum)); the goodness-of-fit represented by the percentage of the variation of Y explained by the model (R2); and the predictive performance (Q2). Permutation testing was performed to validate the OPLS-DA model [37,38]. The percentage of the variation of the dataset predicted by the model (Permuted Q2) was assessed using a cross-validation test [39,40]. Sevenfold cross-validation analysis of variance (CV-ANOVA) was utilized to determine OPLS-DA model significance [38]. Additionally, response permutation testing was performed to validate the OPLS-DA model [37,38]. To this end, the intervention is permutated to appear in a different order while the metabolite-dataset remains intact. Next, a model is then fit to the permutated data. The goodness-of-fit (R2) and predictive performance (Q2) of the permutated model are contrasted to the actual model. A valid model has lower permutated Q2 values compared to the actual model

and a Q2-intercept below zero. A Q2-intercept below zero indicates that the model is stable and non-random, firmly supporting model validity [41].

For day 0, 3 and 7 repeated measures data, correlations between individual metabolites and absolute increase in 25(OH)D levels from day 0 to day 3 over time were determined separately in women and in men utilizing sex-stratified linear mixed effects models correcting for age, baseline 25(OH)D, absolute increase in 25(OH)D at day 3, SAPS II, plasma day, admission diagnosis, and an individual subject-specific random-intercept. A false discovery rate adjusted p-value (q-value) threshold of 0.10 was used to identify all significant differences [34]. Additionally, we performed a subanalysis in a responder cohort of patients who received high dose vitamin D_3 intervention and had an increase in 25(OH)D ≥ 7.5 ng/ml from day 0 to day 3. In day 0, 3 and 7 repeated measures data we determined correlations between individual metabolites over time relative to sex (as the exposure) using linear mixed effects models correcting for age, baseline 25(OH)D, SAPS II, plasma day, admission diagnosis, and an individual subject-specific random-intercept. All mixed models were analyzed using STATA 16.1MP (College Station, TX). For data visualization purposes, rain plots were produced in R-3.6.2 [42].

Lastly, we evaluated a potential sex-specific mediating effect on the association between the absolute increase in 25(OH)D levels from day 0 to day 3 and individual metabolite abundance adjusted for age, baseline 25(OH)D, SAPS II and admission diagnosis. Analyses were performed on each of the 983 metabolites at day 3 using the R package mediation [43] to obtain bootstrap P values (N = 2000 samples) for the

mediation effect of sex. Significant mediation was present if the p value was < 0.01 and 10% or more of the association was mediated through sex [44].

Supplemental Methods References Cited

1. Amrein, K. *et al.* Effect of high-dose vitamin D3 on hospital length of stay in critically ill patients with vitamin D deficiency: the VITdAL-ICU randomized clinical trial. *JAMA* **312**, 1520-30 (2014).
2. Deyo, R.A., Cherkin, D.C. & Ciol, M.A. Adapting a clinical comorbidity index for use with ICD-9-CM administrative databases. *J Clin Epidemiol* **45**, 613-9 (1992).
3. Le Gall, J.R., Lemeshow, S. & Saulnier, F. A new Simplified Acute Physiology Score (SAPS II) based on a European/North American multicenter study. *JAMA* **270**, 2957-63 (1993).
4. Wehrens, R. *et al.* Improved batch correction in untargeted MS-based metabolomics. *Metabolomics* **12**, 88 (2016).
5. Trezzi, J.P. *et al.* Metabolic profiling of body fluids and multivariate data analysis. *MethodsX* **4**, 95-103 (2017).
6. Bain, J.R. *et al.* Metabolomics applied to diabetes research: moving from information to knowledge. *Diabetes* **58**, 2429-43 (2009).
7. Parsons, H.M., Ekman, D.R., Collette, T.W. & Viant, M.R. Spectral relative standard deviation: a practical benchmark in metabolomics. *Analyst* **134**, 478-85 (2009).
8. Narvaez-Rivas, M. & Zhang, Q. Comprehensive untargeted lipidomic analysis using core-shell C30 particle column and high field orbitrap mass spectrometer. *J Chromatogr A* **1440**, 123-134 (2016).
9. Michopoulos, F., Lai, L., Gika, H., Theodoridis, G. & Wilson, I. UPLC-MS-based analysis of human plasma for metabonomics using solvent precipitation or solid phase extraction. *J Proteome Res* **8**, 2114-21 (2009).
10. Want, E.J., Smith, C.A., Qin, C., Van Horne, K.C. & Siuzdak, G. Phospholipid capture combined with non-linear chromatographic correction for improved serum metabolite profiling. *Metabolomics* **2**, 145-154 (2006).
11. Oresic, M., Vidal-Puig, A. & Hanninen, V. Metabolomic approaches to phenotype characterization and applications to complex diseases. *Expert Rev Mol Diagn* **6**, 575-85 (2006).
12. Chen, W.W., Freinkman, E., Wang, T., Birsoy, K. & Sabatini, D.M. Absolute Quantification of Matrix Metabolites Reveals the Dynamics of Mitochondrial Metabolism. *Cell* **166**, 1324-1337 e11 (2016).
13. Hufsky, F., Scheubert, K. & Bocker, S. Computational mass spectrometry for small-molecule fragmentation. *TrAC Trends Anal. Chem.* **53**, 41–48 (2014).
14. Dunn, W.B. *et al.* Procedures for large-scale metabolic profiling of serum and plasma using gas chromatography and liquid chromatography coupled to mass spectrometry. *Nat Protoc* **6**, 1060-83 (2011).
15. Sumner, L.W. *et al.* Proposed minimum reporting standards for chemical analysis Chemical Analysis Working Group (CAWG) Metabolomics Standards Initiative (MSI). *Metabolomics* **3**, 211-221 (2007).
16. Schrimpe-Rutledge, A.C., Codreanu, S.G., Sherrod, S.D. & McLean, J.A. Untargeted Metabolomics Strategies-Challenges and Emerging Directions. *J Am Soc Mass Spectrom* **27**, 1897-1905 (2016).

17. Guasch-Ferre, M. *et al.* Plasma acylcarnitines and risk of cardiovascular disease: effect of Mediterranean diet interventions. *Am J Clin Nutr* **103**, 1408-16 (2016).
18. Weljie, A.M., Newton, J., Mercier, P., Carlson, E. & Slupsky, C.M. Targeted profiling: quantitative analysis of 1H NMR metabolomics data. *Anal Chem* **78**, 4430-42 (2006).
19. Wishart, D.S. Quantitative metabolomics using NMR. *TrAC Trends Anal. Chem.* **27**, 228–237 (2008).
20. Zhou, B., Xiao, J.F., Tuli, L. & Ressom, H.W. LC-MS-based metabolomics. *Mol Biosyst* **8**, 470-81 (2012).
21. Parisi, L.R., Li, N. & Atilla-Gokcumen, G.E. Very Long Chain Fatty Acids Are Functionally Involved in Necroptosis. *Cell Chem Biol* **24**, 1445-1454 e8 (2017).
22. Junot, C., Madalinski, G., Tabet, J.C. & Ezan, E. Fourier transform mass spectrometry for metabolome analysis. *Analyst* **135**, 2203-19 (2010).
23. Kamleh, A. *et al.* Metabolomic profiling using Orbitrap Fourier transform mass spectrometry with hydrophilic interaction chromatography: a method with wide applicability to analysis of biomolecules. *Rapid Commun Mass Spectrom* **22**, 1912-8 (2008).
24. Kamleh, M.A., Hobani, Y., Dow, J.A. & Watson, D.G. Metabolomic profiling of Drosophila using liquid chromatography Fourier transform mass spectrometry. *FEBS Lett* **582**, 2916-22 (2008).
25. Koulman, A. *et al.* High-resolution extracted ion chromatography, a new tool for metabolomics and lipidomics using a second-generation orbitrap mass spectrometer. *Rapid Commun Mass Spectrom* **23**, 1411-8 (2009).
26. Xiao, J.F., Zhou, B. & Ressom, H.W. Metabolite identification and quantitation in LC-MS/MS-based metabolomics. *Trends Analyt Chem* **32**, 1-14 (2012).
27. Seamans, K.M. & Cashman, K.D. Existing and potentially novel functional markers of vitamin D status: a systematic review. *Am J Clin Nutr* **89**, 1997S-2008S (2009).
28. Oliver, S.G., Winson, M.K., Kell, D.B. & Baganz, F. Systematic functional analysis of the yeast genome. *Trends Biotechnol* **16**, 373-8 (1998).
29. Amrein, K., Lasky-Su, J.A., Dobnig, H. & Christopher, K.B. Metabolomic basis for response to high dose vitamin D in critical illness. *Clin Nutr* **40**, 2053-2060 (2021).
30. Chary, S., Amrein, K., Lasky-Su, J., Dobnig, H. & Christopher, K.B. The Sex-specific Metabolic Response to Critical Illness: a post-hoc metabolomics study of the VITdAL-ICU trial. *Scientific Reports* **11**, 3951 (2021).
31. van den Berg, R.A., Hoefsloot, H.C., Westerhuis, J.A., Smilde, A.K. & van der Werf, M.J. Centering, scaling, and transformations: improving the biological information content of metabolomics data. *BMC Genomics* **7**, 142 (2006).
32. Struja, T. *et al.* Metabolomics for Prediction of Relapse in Graves' Disease: Observational Pilot Study. *Front Endocrinol (Lausanne)* **9**, 623 (2018).
33. Chong, J. & Xia, J. Using MetaboAnalyst 4.0 for Metabolomics Data Analysis, Interpretation, and Integration with Other Omics Data. *Methods Mol Biol* **2104**, 337-360 (2020).
34. Benjamini, Y. & Yekutieli, D. The control of the false discovery rate in multiple testing under dependency. *Ann Stat* **29**, 1165-1188 (2001).

35. Bylesjö, M. *et al.* OPLS discriminant analysis: combining the strengths of PLS-DA and SIMCA classification. *Journal of Chemometrics* **20**, 341–351 (2006).
36. Trygg, J. & Wold, S. Orthogonal projections to latent structures (O-PLS). *J. Chemometrics* **16**, 119–128 (2002).
37. Westerhuis, J.A. *et al.* Assessment of PLSDA cross validation. *Metabolomics* **4**, 81–89 (2008).
38. Eriksson, L., Trygg, J. & Wold, S. CV-ANOVA for significance testing of PLS and OPLS models. *Journal of Chemometrics* **22**, 594-600 (2008).
39. Eastment, H. & Krzanowski, W. Crossvalidatory choice of the number of components from a principal component analysis. *Technometrics* **24**, 73-77 (1982).
40. Martens, H. & Naes, T. *Multivariate Calibration*, 504 (John Wiley and Sons, Chichester, 1989).
41. Triba, M.N. *et al.* PLS/OPLS models in metabolomics: the impact of permutation of dataset rows on the K-fold cross-validation quality parameters. *Mol Biosyst* **11**, 13-9 (2015).
42. Henglin, M. *et al.* A Single Visualization Technique for Displaying Multiple Metabolite-Phenotype Associations. *Metabolites* **9**(2019).
43. Dustin, T., Yamamoto, T., Hirose, K., Keele, L. & Imai, K. mediation: R package for causal mediation analysis. *Journal of Statistical Software* **59**, 1-38 (2014).
44. Pietzner, M. *et al.* Hepatic Steatosis Is Associated With Adverse Molecular Signatures in Subjects Without Diabetes. *J Clin Endocrinol Metab* **103**, 3856-3868 (2018).
