# Supplementary material for: Sex-Specific Catabolic Metabolism Alterations in the Critically Ill following High Dose Vitamin D
Source: Metabolites. 2022 Feb 25;12(3):207. doi: 10.3390/metabo12030207 (PMC8953844; doi:10.3390/metabo12030207)
Supplement: Supplementary file 1 [file metabolites-12-00207-s001.zip › Table S1.pdf]

Table S1. Pharmacokinetics of High Dose Vitamin D3

Parameter	Women	Men	P-value
N	67	108	
Dose/kg body weight IU/kg Mean (SD)	7973 (1703)	7788 (1612)	<0.001
C0 ng/ml Mean (SD)	12.47 (4.89)	12.79 (4.50)	0.66
C3 ng/ml Mean (SD)	32.63 (19.63)	33.78 (17.52)	0.69
C7 ng/ml Mean (SD)	35.78 (22.18)	35.50 (20.60)	0.93
AUC0-7 (ng.h/ml)	4850.67 (2714.36)	4965.21 (2453.75)	0.77
AUCnorm (ng.h/ml)/(IU/kg) Median [IQR]	0.57 [0.34, 0.80]	0.67 [0.48, 0.99]	0.034
