# Supplementary material for: Sex-Specific Catabolic Metabolism Alterations in the Critically Ill following High Dose Vitamin D
Source: Metabolites. 2022 Feb 25;12(3):207. doi: 10.3390/metabo12030207 (PMC8953844; doi:10.3390/metabo12030207)
Supplement: Supplementary file 1 [file metabolites-12-00207-s001.zip › Table S3.pdf]

Table S3. OPLS-DA model goodness of fit and predictive ability

Characteristic	OPLS-DA			Permutation (*n*=200)		CV-ANOVA
	R2X	R2Y	Q2	R2 intercept (*x*-axis, *y*-axis)	Q2 intercept (*x*-axis, *y*-axis)	P-value
25(OH)D ≥ or < 7.5 ng/ml in women	0.107	1.00	-0.109	0.00, 0.229	0.00, -0.153	0.99
25(OH)D ≥ or < 7.5 ng/ml in men	0.112	1.00	-0.0256	0.00, 0.132	0.00, -0.128	0.99
