# Supplementary figures and images for: Sex-Specific Catabolic Metabolism Alterations in the Critically Ill following High Dose Vitamin D
Source: Metabolites. 2022 Feb 25;12(3):207. doi: 10.3390/metabo12030207 (PMC8953844; doi:10.3390/metabo12030207)

Figure S1: Day 0, 3 and 7 serum 25(OH)D levels in Women and Men

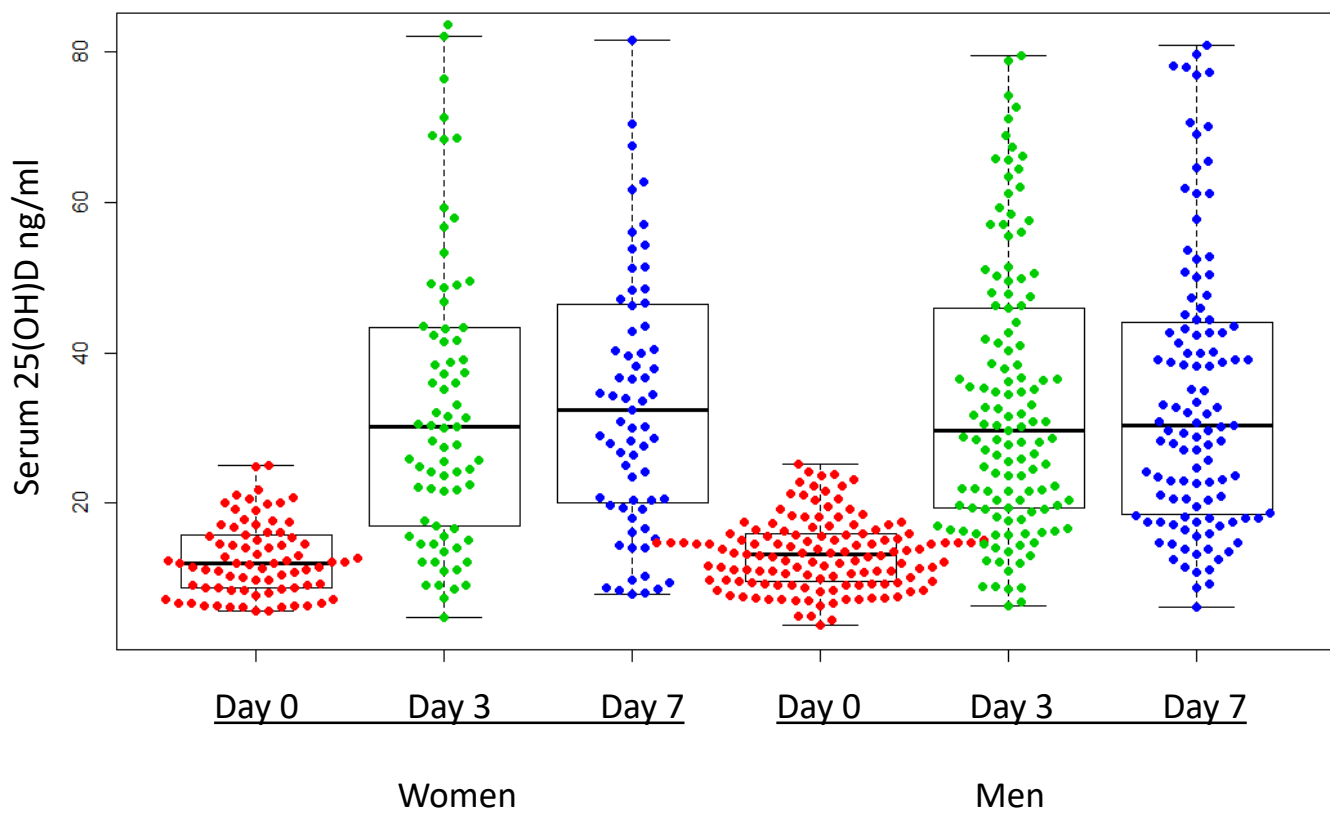

Supplement: Supplementary file 1 [file metabolites-12-00207-s001.zip › Figure S1.pdf]

Figure S2: Responder Cohort Short-Chain Acylcarnitines

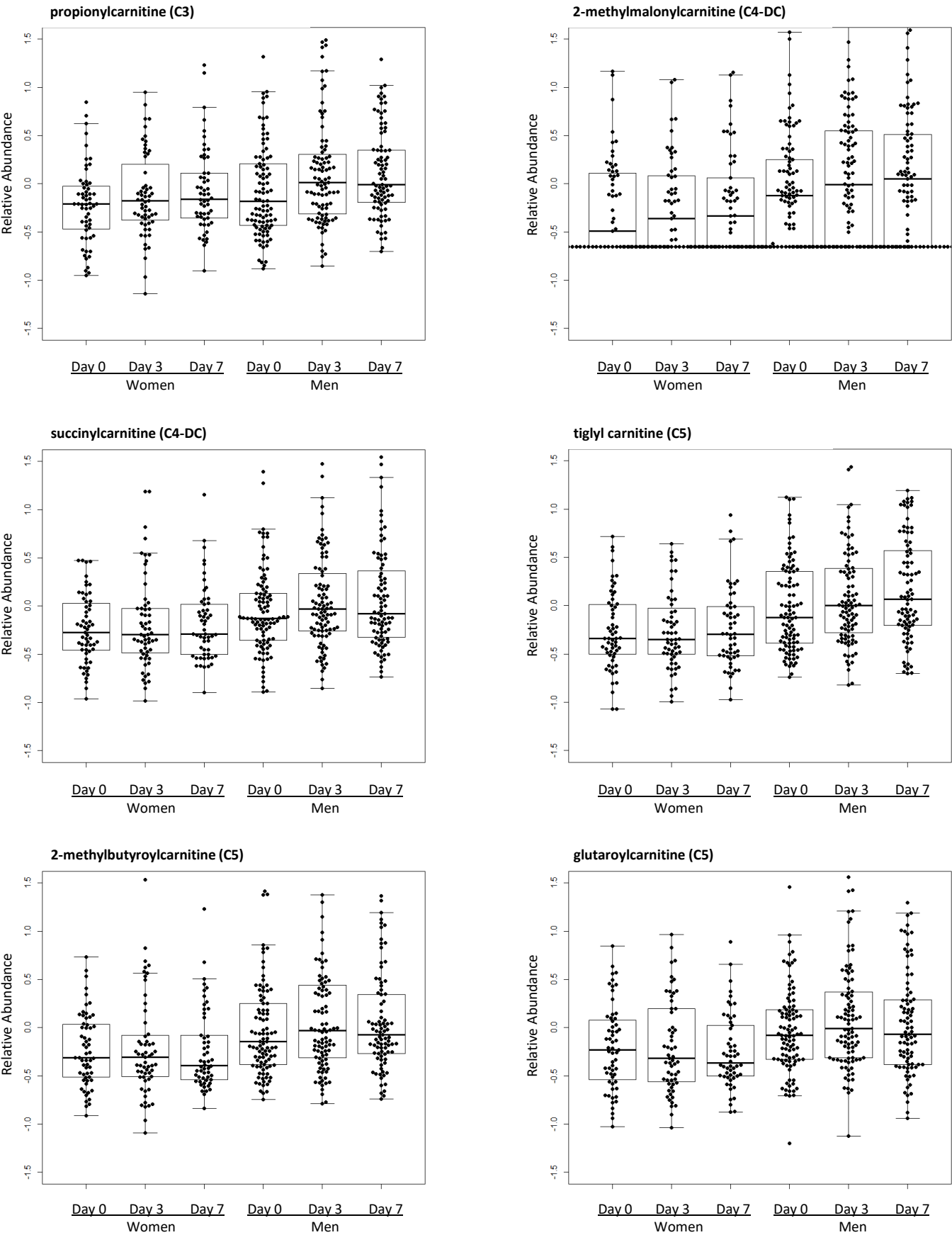

Supplement: Supplementary file 1 [file metabolites-12-00207-s001.zip › Figure S2.pdf]

Figure S3: Responder Cohort BCAA Metabolites

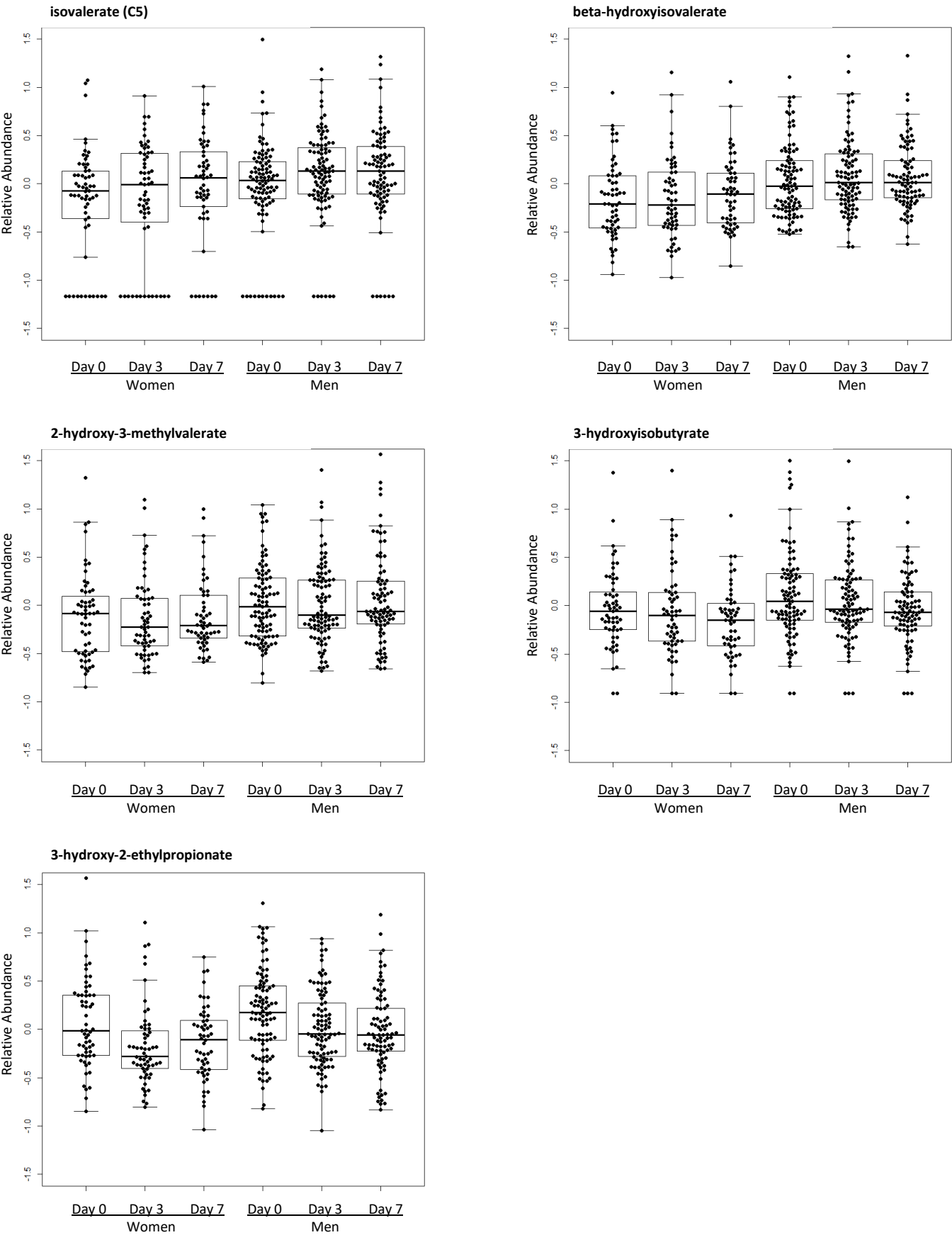

Supplement: Supplementary file 1 [file metabolites-12-00207-s001.zip › Figure S3.pdf]
